# Coconut Water Microfiltration Optimization Using Response Surface Modeling, Neural Networks, and Genetic Algorithms: Performance and Nutritional Retention

**DOI:** 10.3390/membranes16070221

**Published:** 2026-06-26

**Authors:** José Diogo da Rocha Viana, Arthur Claudio Rodrigues de Souza, Paulo Riceli Vasconcelos Ribeiro, Lorena Mara Alexandre Silva, Kirley Marques Canuto, Katia Rezzadori, Giordana Demaman Arend, Ana Paula Dionísio, José Carlos Cunha Petrus

**Affiliations:** 1 Department of Chemical and Food Engineering, Federal University of Santa Catarina, Florianópolis 88040-900, SC, Brazil; diogo.rocha@posgrad.ufsc.br (J.D.d.R.V.); jose.petrus@ufsc.br (J.C.C.P.); 2 Embrapa Tropical Agroindustry, Fortaleza 60511-110, CE, Brazil; arthur.souza@embrapa.br (A.C.R.d.S.); paulo.riceli@embrapa.br (P.R.V.R.); lorena.mara@embrapa.br (L.M.A.S.); kirley.canuto@embrapa.br (K.M.C.); 3 Department of Food Science and Technology, Federal University of Santa Catarina, Av. Ademar Gonzaga, 1346, Itacorubi, Florianópolis 88034-000, SC, Brazil; katia.rezzadori@ufsc.br

**Keywords:** *Cocos nucifera* L., crossflow microfiltration, response surface methodology, membrane fouling, nuclear magnetic resonance (NMR), nutritional retention

## Abstract

Although coconut water is recognized for its desirable sensory appeal and nutritional composition, its broader industrial use is constrained by the rapid deterioration that occurs after extraction. In this study, crossflow microfiltration of coconut water with a silicon carbide membrane was optimized by investigating pressure and temperature through a face-centered design (FCD) and artificial neural network modeling coupled with a genetic algorithm (ANN–GA). Permeate flux and fouling index were used as process responses, and the optimized condition was further examined in terms of hydraulic resistance, fouling behavior, and retention of minerals and primary metabolites. Pressure and temperature affected the process differently: permeate flux showed marked nonlinear behavior, whereas fouling index was governed mainly by pressure. At the sample level, ANN described permeate flux more accurately than FCD (R^2^ = 0.99 vs. 0.96), whereas FCD showed better grouped cross-validated predictivity across unseen pressure–temperature conditions (Q^2^ = 0.85 vs. 0.57). For the fouling index, FCD outperformed ANN in both sample-level fit and grouped validation (R^2^ = 0.95 vs. 0.60; Q^2^ = 0.70 vs. 0.61). Both approaches converged on the same favorable operating window, and experimental validation at 60 kPa and 35 °C yielded 1085.23 ± 23.12 L h^−1^ m^−2^ and 83.56 ± 1.56%. During concentration mode, flux decline was severe but predominantly reversible, with high clean-water permeance recovery after chemical cleaning. Resistance partition and fouling modeling indicated that the main hydraulic limitation was associated with concentration polarization and external cake-layer buildup rather than irreversible membrane damage. The clarified fraction also preserved high transmission of major minerals and relevant primary metabolites, indicating that the selected condition combined high productivity, manageable fouling, and satisfactory nutritional retention.

## 1. Introduction

Coconut water is valued for its fresh sensory profile and for its composition, which includes soluble carbohydrates, minerals, amino acids, and other low-molecular-weight compounds [1,2]. Despite these attributes, its industrial use is still limited by rapid spoilage. After extraction, coconut water is prone to microbial growth, enzymatic activity, and chemical reactions that can quickly affect color, flavor, and overall quality [1,3,4,5,6]. Thermal processing can improve preservation, but it may also affect sensory properties and part of the beverage’s native chemical composition. This has kept interest in milder preservation methods that are able to maintain characteristics closer to those of fresh coconut water [3,4,5,6].

Crossflow microfiltration is one of these alternatives. The process can clarify the beverage under relatively mild conditions, reduce suspended material and microbial load, and better preserve heat-sensitive compounds when compared with conventional high-temperature treatments [7,8,9]. In coconut water, this aspect is especially important because product value is not based only on shelf-life extension. It also depends on maintaining the dissolved compounds that define its nutritional and compositional profile [1,6,9]. Therefore, microfiltration should not be evaluated only in terms of hydraulic performance. It is also necessary to verify whether the clarified fraction remains compositionally close to the original beverage, mainly regarding primary metabolites and minerals.

Fouling remains the main challenge for stable membrane operation [10,11]. In fruit-based liquids, flux decline is commonly related to concentration polarization, pore blocking, and deposit formation on the membrane surface. The relative importance of each phenomenon depends on feed composition, membrane characteristics, and operating conditions [10,11,12]. Pressure and temperature are particularly relevant because both affect mass transfer and fouling development. Higher pressure can increase the initial driving force for permeation, but it may also intensify deposit compaction and cause greater permeability losses. Temperature affects viscosity and transport behavior, with direct effects on permeation and on the formation of the polarized layer [10,11,13]. For this reason, suitable operating conditions should balance productivity and hydraulic stability instead of simply maximizing flux. In this context, the critical flux concept is still useful as a qualitative reference for identifying the transition toward more severe fouling conditions [12].

The choice of membrane material also influences process performance. Polymeric modules are widely used in liquid-food processing because they are less expensive, but ceramic membranes become more suitable when mechanical strength, chemical resistance, and repeated cleaning are important requirements [11,14,15]. Among them, silicon carbide (SiC) stands out for its high permeability, low tortuosity, and resistance to aggressive thermal and chemical cleaning [14,16]. These properties are especially relevant in fouling-prone systems that require recurrent clean-in-place operation. In coconut water, previous work performed under matched hydrodynamic conditions showed that SiC delivered higher permeate flux, lower energy demand, and smaller compositional changes in the clarified fraction than ceramic alumina and polymeric polypropylene modules [17]. Other studies on coconut-water microfiltration also supported SiC as a technically appropriate membrane platform [18]. Accordingly, the present work did not revisit membrane-material screening; instead, it used SiC as the selected platform for a focused optimization of operating conditions.

Response surface methodology provides an interpretable starting point for studying the effects and interactions of operating variables within a delimited experimental region [19,20,21]. Still, membrane responses are not always well represented by a simple quadratic surface, particularly when flux and fouling emerge from coupled nonlinear transport processes [11,22]. Artificial neural networks can complement this approach by learning input–output behavior without imposing a predefined polynomial form [22,23], and, when linked to genetic algorithms, they can also serve as surrogate models for numerical optimization [23,24]. In compact experimental domains such as the present one, however, ANN–GA should be viewed as complementary to FCD/RSM rather than as an automatic substitute. Its relevance lies in testing whether an independent nonlinear framework converges to the same favorable operating region. Comparative use of these two strategies remains limited in coconut-water microfiltration [18]. Accordingly, this work investigated the optimization of coconut-water crossflow microfiltration with a silicon carbide membrane by examining pressure and temperature through a face-centered design together with artificial neural network modeling integrated with a genetic algorithm. In addition to optimizing the operating condition, the study compares the modeling approaches, experimentally validates the selected condition, examines hydraulic resistance, interprets fouling during constant-pressure filtration, and evaluates the retention of minerals and primary metabolites in the clarified fraction.

## 2. Materials and Methods

### 2.1. Raw Material and Sample Preparation

Green coconut water was supplied by Nosso Coco (Paraipaba Agroindustrial Ltd.a., Paraipaba, Brazil). According to the supplier, the commercial product already contained potassium metabisulfite (K_2_S_2_O_5_; approximately 10 mg L^−1^), and no additional metabisulfite was added by the authors. Potassium metabisulfite is commonly used during coconut-water handling and storage to reduce oxidative browning and pink discoloration [25].

### 2.2. Process Optimization Design

A two-factor face-centered design (FCD) was adopted to optimize coconut-water microfiltration as a function of transmembrane pressure (kPa) and temperature (°C). The design comprised 13 coded experiments, including five center points. In terms of unique settings, the FCD represented nine pressure–temperature conditions; however, 29 independent runs were carried out because each non-center condition was performed in triplicate and the center condition in quintuplicate. This replication enabled estimation of within-condition variability and supported sample-level comparison between models.

All experiments were conducted with a single industrial batch of green coconut water from the same manufacturer. After receipt, the batch was portioned into twenty-nine 6.0 L packages and kept refrigerated until use. For each run, one package was processed independently. Therefore, the variability reported in Table 1 represents between-run experimental variation within a single batch rather than repeated analytical measurements of the same permeate sample. The experiments were performed with a SiC membrane having an average pore diameter of 0.6 µm.

The responses modeled in the design were permeate flux (L h^−1^ m^−2^) and fouling index (%). Pressure and temperature were chosen because they were the two continuously adjustable variables of the pilot unit with direct influence on driving force, viscosity-related transport, and fouling development. By contrast, crossflow velocity was fixed at 6 m s^−1^ to maintain a constant hydrodynamic regime, while feed composition and pH were kept outside the design because all assays used one homogenized industrial batch and the study addressed operational optimization rather than feed conditioning. The investigated window (50–200 kPa; 20–40 °C) was selected to span a practical range of the pilot system and the SiC membrane while still generating measurable changes in flux and fouling index without destabilizing operation. The practical relevance of this low-pressure region is consistent with previous coconut-water microfiltration studies that reported economic feasibility around 0.65 bar [26].

On this basis, a face-centered design was selected because it concentrates the coded levels at the lower limit (−1), center (0), and upper limit (+1) of the experimental window (Figure S1; Supplementary Materials). The tested levels are summarized in Table 1.

#### 2.2.1. Face-Centered Response Surface Modeling

FCD data were modeled with a second-order polynomial (Equation (1)) including linear, quadratic, and interaction terms for the two independent variables. In this formulation, *Y* denotes the response, *X_i_* and *X_j_* the coded factors, $\beta_{0}$ the intercept, $\beta_{i}$ the linear coefficients, $\beta_{ii}$ the quadratic coefficients, and $\beta_{ij}$ the interaction coefficients [27]. The experimental design and ANOVA calculations were performed on the Protimiza Experimental Design web platform [28], using *p* < 0.05 as the significance threshold.

(1) $$Y=\beta_{0}+\sum_{i=1}^{j}\beta_{i}X_{j}+\sum_{i=1}^{j}\beta_{ii}X_{i}^{2}+\sum_{i\neq j=1}^{j}\beta_{ij}X_{i}X_{j}\;$$

#### 2.2.2. ANN Modeling and GA-Based Optimization

**Figure 1:**
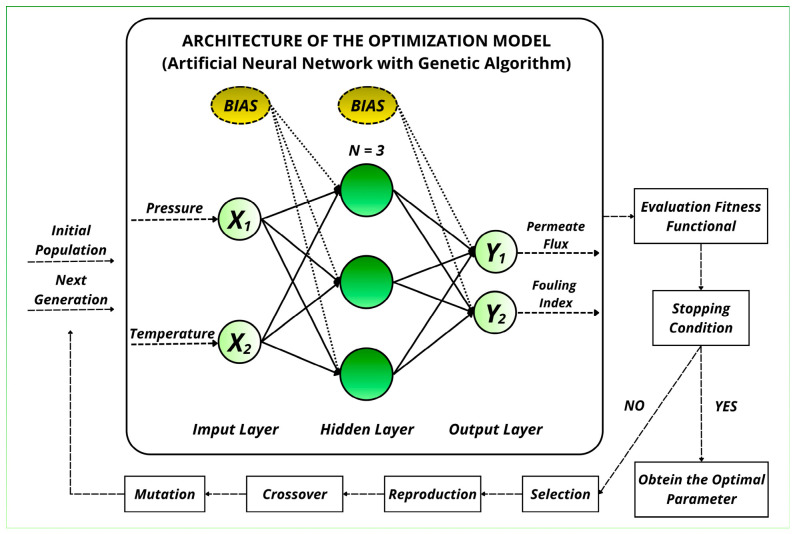
Workflow used for ANN construction and GA-driven response optimization.

After ANN training, optimization was performed separately for each response using a genetic algorithm within the lower and upper bounds of the experimental domain. For the permeate-flux response, optimization was carried out by minimizing the negative value of the ANN estimate (*fitness* = −*J_p_*), which allowed the search procedure to treat flux maximization within MATLAB’s (version R2024b) native minimization scheme. For the fouling index, the objective function was the ANN-predicted fouling index itself (*fitness* = *FI*), which was directly minimized. Thus, the optimization was not treated as a formal weighted multiobjective problem; instead, each response was optimized independently. The genetic algorithm operated with 120 candidate solutions per generation, a total of 50 generations, 5% elitism, adaptive feasible mutation, and an early-stopping criterion of 8 stall generations. This ANN-GA strategy allows nonlinear response exploration without requiring an explicit polynomial equation, which is advantageous when the response surface may present non-uniform local behavior [29,30,31]. A schematic representation of the ANN-GA modeling and optimization workflow is presented in Figure 1.

Prior to training, input and output matrices were processed with *removeconstantrows* and normalized by *mapminmax* to standardize scale and improve numerical conditioning during training [32,33]. The ANN structure comprised two input nodes (pressure and temperature), one hidden layer, and two output nodes (permeate flux and fouling index). Because the dataset was limited, model selection favored parsimony. The number of hidden neurons was determined empirically from repeated runs by choosing the configuration with the best validation mean squared error while avoiding unnecessarily large networks; this procedure led to a 2–3–2 topology. Training was performed with Bayesian regularization backpropagation (*trainbr*), using mean squared error as the performance criterion, because this algorithm is suitable for small datasets and helps restrain excessive weight growth [33]. For diagnostic plots, the full dataset was split randomly at the sample level into training, validation, and test subsets in a 70/15/15 proportion. Twenty networks were trained independently under the same settings, and the model with the lowest validation MSE was retained for contour plotting, response-surface generation, and coupling with the GA. The hidden and output layers used the *tansig* and *purelin* transfer functions, respectively (Equations (2) and (3)) [30].

(2) tansig n=1[1+e−2n]−1

(3) $$purelin\;\left(n\right)=n$$

Predictive robustness beyond the internal random split was examined with leave-one-condition-out grouped cross-validation. In each fold, all replicates belonging to one pressure–temperature setting were excluded from calibration and used only for external prediction. This grouped scheme is stricter than sample-wise random splitting because it prevents replicate leakage and better reflects prediction at unseen operating conditions [34]. Within each fold, ANN prediction was obtained from a bagged ensemble of 25 Bayesian-regularized networks. Bootstrap resampling was applied to the calibration subset; each network was fitted with *dividetrain*, and the estimate for the withheld condition was calculated as the average ensemble output [35].

Accordingly, the grouped cross-validation results reported in this study were based on aggregated ensemble predictions rather than on a single trained network, which improves reproducibility and reduces sensitivity to any specific initialization. For comparison, a quadratic response surface model including linear, interaction, and squared terms was fitted under the same cross-validation structure. In the present dataset, this procedure was applied to 29 observations distributed across 9 unique pressure-temperature conditions.

### 2.3. Crossflow Microfiltration Configuration and Operating Conditions

Three crossflow microfiltration arrangements equipped with silicon carbide modules (SiC, Crystar™ FT600, Cavaillon, France) were evaluated. Figure S2 (Supplementary Materials) summarizes the system layouts and membrane specifications, including the average pore size of 0.6 µm. To allow comparison among configurations, transmembrane pressure, bulk temperature, and crossflow velocity were kept fixed, and all performance data were normalized by total area (L h^−1^ m^−2^). After each clean-in-place (CIP) cycle, clean-water flux recovery remained at or above 95% for all membranes.

All assays were carried out on the same pilot unit, composed of a 10 L feed tank and a NEMO 1.0 CV (Pomerode, Brazil) progressive-cavity pump. The combined dead volume of the pump, tubing, and membrane module was approximately 1.0 L. The tangential microfiltration processes were carried out at ΔP_TM_ = 60 kPa and 35 °C. Two operating modes were employed. The runs used for FCD and ANN modeling were conducted under total recirculation (VRR = 1), with both permeate and retentate returned to the feed tank so that the working volume remained essentially constant. After the operating point had been selected, separate concentration-mode assays (VRR > 1) were performed to evaluate flux evolution, hydraulic resistances, fouling behavior, and the transmission/rejection of minerals and primary metabolites.

The nominal tangential velocity (*v_t_*) inside the channel was set to 6 m s^−1^ by adjustment of pump speed. This value was estimated from the recirculation flow rate and representative channel geometry rather than measured directly in the channel. Based on this imposed setpoint, Reynolds number (*Re*), wall shear stress (*τ_w_*), and an order-of-magnitude mass-transfer coefficient (*k*) were calculated using Equations (4)–(9) and are reported in Table 2.

(4) $$Re=\frac{\rho v_{t}d_{h}}{\mu }$$

(5) $$Sc=\frac{\mu }{\rho D_{m}}$$

(6) $$f=0.3164\;{Re}^{-0.25}$$

(7) $$\tau_{w}=\frac{f}{8}\;\rho v_{t}^{2}$$

(8) $$Sh=0.023\;{Re}^{0.83}\;{Sc}^{0.33}$$

(9) $$k=\frac{S_{h}D_{ab}}{d_{h}}$$

where “*Re*”, “*Sc*”, and “*Sh*” are the Reynolds, Schmidt, and Sherwood numbers, respectively; “*f*” is the Darcy friction factor; *τ_w_* is the wall shear stress; and “*k*” is the mass-transfer coefficient. The terms ρ, *μ*, *v_t_*, *d_h_*, and *D_ab_* represent fluid density, dynamic viscosity, in-channel tangential velocity, hydraulic diameter, and representative solute diffusivity, respectively.

Table 2 shows that the imposed 6.0 m s^−1^ condition corresponded to *Re* = 2.99 × 10^4^, *τ_w_* = 107.7 Pa, and *k* = 2.71 × 10^−4^ m s^−1^, which characterizes a turbulent and strongly convective regime. Under these conditions, crossflow velocity was treated as a fixed hydrodynamic background variable rather than as an optimization factor, allowing the specific effects of pressure and temperature on flux and fouling index to be evaluated more directly.

#### 2.3.1. Calculation of Process Performance Parameters

Permeate flux, volumetric retention ratio, transmission, and retention were obtained from Equations (10)–(13). In these calculations, *J_p_* was updated every 2 min from the collected permeate volume (*V_P_*), membrane area (*A_m_*), and collection time (*t*). VRR was calculated from feed and retentate volumes (*V_F_* and *V_R_*), whereas transmission and retention were expressed as the ratio between permeate and feed solute concentrations (*C_P_* and *C_A_*, mg L^−1^).

(10) JP=VPt×Am

(11) VRR=VfVr

(12) T=CPCA×100

(13) R=1−CPCA×100

#### 2.3.2. Hydraulic Resistance and Fouling Analysis

Hydraulic resistances and permeance coefficients were computed according to the literature using Equations (14)–(24), summarized in Table 3.

As an additional interpretive reference, the critical-flux concept was used qualitatively to relate resistance distribution to operating conditions [39,40].

The predominant fouling mechanism was analyzed with the models originally proposed by Hermia [41] and later adapted by Bolton [42]. These formulations account for complete blocking, intermediate blocking, standard blocking, cake filtration, and combinations thereof, as summarized in Table 4.

### 2.4. Membrane Cleaning Procedure

The cleaning procedure was established from preliminary screening tests carried out after coconut-water filtration, together with manufacturer guidance, with the aim of restoring permeability without damaging the membrane. For the SiC module, the chemical-cleaning temperature was set at 80 °C according to the manufacturer’s recommendation, while the contact times adopted for recirculation and backwashing were those that provided satisfactory permeability recovery in the preliminary tests. The protocol consisted of total recirculation of 3.0% (*w*/*v*) NaOH plus 1.0% (*w*/*v*) NaClO for 10 min at 80 °C, followed by alkaline backwashing with the same solution for another 10 min at 80 °C. After rinsing to pH 7.0, a second recirculation step was performed with 1.0% (*w*/*v*) HNO_3_ for 20 min, followed by a final rinse with deionized water (conductivity < 10 µS) until neutral pH [18]. Cleaning efficiency was expressed as the ratio of post-CIP to initial clean-water flux; values above 95% were taken as evidence of minimal residual irreversible fouling and adequate comparability among membranes.

### 2.5. NMR-Based Identification and Quantification of Organic Compounds

For NMR analysis, each sample was prepared by mixing 160 µL of coconut water with 400 µL of D_2_O (99.9%) and 40 µL of a D_2_O solution containing 14 mM EDTA and 1% sodium 3-(trimethylsilyl)propionate-d4 (TMSP-d4) as internal standard, followed by transfer to 5 mm tubes. EDTA was included to reduce spectral shifts associated with ionic strength, since coconut water is naturally rich in mineral salts [6,17]. Spectra were acquired on an Agilent 600 MHz spectrometer equipped with a 5 mm inverse-detection probe (^1^H/^19^F/^15^N/^31^P). Quantitative ^1^H NMR experiments were performed in triplicate using PRESAT to suppress residual water at δ 4.86 (Figure S3 in Supplementary Materials). Acquisition conditions were 25 °C, 8.2 µs 90° pulse at 58 dB, 5.0 s acquisition time, and 15 s relaxation delay, corresponding to 7T1 and 99.9% relaxation accuracy [43,44]. A spectral window of 16 ppm and a fixed receiver gain of 40 were applied to all samples. Before the Fourier transformation, the free induction decays were apodized with an exponential function equivalent to 0.3 Hz line broadening and processed with 32 k points and phase correction [45].

Compound assignment relied on ^1^H and ^13^C chemical shifts, ^1^H–^13^C correlations, and ^1^H coupling constants obtained from COSY, HSQC, and HMBC experiments, combined with open-access databases and literature information (Table S1 in Supplementary Materials) [4,5,6,17].

Signals free of spectral overlap were quantified with the external-reference procedure available in VnmrJ™ (version 4.2, Agilent, Santa Clara, CA, USA). A 5.0 mg L^−1^ sucrose solution was used to calibrate the spectrometer, and the probe profile was updated with the parameters required for concentration measurements [6,17]. Compound concentrations were then obtained from Equation (34), in which *P_X_* is the concentration of compound *X*, *P_std_* the concentration of the external standard, *I_X_* and *_Istd_* the integrated signal areas, *N_X_* and *N_std_* the numbers of contributing nuclei, *M_X_* and *M_std_* the molecular masses, and *m_X_* and mstd the sample and standard masses, respectively [44,46].

(34) PX=IXIstd×NstdNX×MXMstd×mstdmX×Pstd

### 2.6. Determination of Mineral Composition

For mineral determination, 1.0 g of the sample was subjected to acid digestion following previously described procedures [18,47]. Each aliquot was placed in a digestion tube, mixed with nitric:perchloric acid (3:1, *v*/*v*), allowed to react for 12 h, and then digested in a dry-block system at 250 °C for 4 h. After cooling, the digests were brought to 50 mL with deionized water and filtered through quantitative paper. Phosphorus, potassium, calcium, magnesium, sodium, manganese, iron, sulfur, zinc, and copper were measured by ICP-OES (Agilent 5100, Mulgrave, Australia).

### 2.7. Statistical Analysis

Model performance was evaluated with *MAD*, *MSE*, *NMSE*, *RMSE*, *NRMSE*, *MAPE*, and *R*^2^, calculated from Equations (35)–(41) [48]. Predictive generalization was examined separately through leave-one-condition-out grouped cross-validation, from which *Q*^2^ and *RMSE_CV_* were obtained (Equations (42) and (43)) [18]. In interpreting these metrics, lower errors indicate better performance, whereas *Q*^2^ and *RMSE_CV_* were prioritized as indicators of robustness to unseen operating conditions; *R*^2^ was used only as a sample-level goodness-of-fit measure. Negative *Q*^2^ values denote predictions poorer than those obtained from the overall experimental mean.

(35) $$MAD=\frac{\sum \left|X_{P}-X_{A}\right|}{n}$$

(36) $$MSE=\frac{\sum {\left(X_{P}-X_{A}\right)}^{2}}{n}$$

(37) $$NMSE=\frac{MSE}{X_{M}}$$

(38) $$MAPE=\frac{100}{n}\sum \left|\frac{X_{A}-X_{P}}{X_{A}}\right|$$

(39) $$RSME=\sqrt{MSE}$$

(40) $$NRSME=\frac{RSME}{X_{M}}$$

(41) $$R^{2}=1-\frac{\sum {\left(X_{P}-X_{A}\right)}^{2}}{\sum {\left(X_{P}-X_{M}\right)}^{2}}$$

(42) $$Q^{2}=1-\frac{\sum {\left(X_{A}-X_{P}^{CV}\right)}^{2}}{\sum {\left(X_{P}-X_{M}\right)}^{2}}$$

(43) $${RMSE}_{CV}=\sqrt{\frac{1}{n}}\sum (X_{A}-X_{P}^{CV})$$

where $X_{P}$ denotes the estimated values generated by the model, $X_{A}$ corresponds to the observed experimental values, $X_{M}$ is the average of the experimental observations, $X_{P}^{CV}$ refers to the prediction obtained for a pressure–temperature condition excluded from model fitting, and n is the total number of experimental runs.

## 3. Results and Discussion

### 3.1. Effects of Pressure and Temperature on Process Responses

The results in Table 1 show that pressure and temperature affected the process differently for the two responses. For permeate flux, their effects were clearly coupled. At 50 kPa, increasing the temperature from 20 to 40 °C raised the flux from 403.84 ± 5.89 to 1247.79 ± 10.97 L h^−1^ m^−2^, whereas at 200 kPa, the same temperature increase reduced the flux from 1083.99 ± 72.93 to 1005.54 ± 15.95 L h^−1^ m^−2^. This indicates that the beneficial effect of temperature, mainly associated with viscosity reduction and improved mass transfer, was progressively limited as pressure increased and the system moved toward a regime more affected by resistance buildup [9,10,11,12,13].

In contrast, the fouling index was governed mainly by pressure. At 20 °C, it increased from 80.88 ± 1.30% at 50 kPa to 93.83 ± 0.78% at 200 kPa; at 30 °C, from 84.65 ± 0.44% to 94.64 ± 0.32%; and at 40 °C, from 83.77 ± 0.47% to 95.16 ± 0.46%. Thus, pressure was not a uniformly favorable factor. Although it increased flux in some conditions, it also made the process hydraulically less favorable. This response agrees with the behavior expected in crossflow membrane filtration, where higher transmembrane pressure increases convective transport toward the membrane surface but also promotes concentration polarization and foulant accumulation [10,11,12]. The buildup of hydraulic resistance and the fouling mechanisms are discussed in more detail in Section 3.2.

For process operation, the most favorable region was not located at the highest pressure. The condition of 50 kPa and 40 °C gave the highest permeate flux while maintaining a moderate fouling index. In contrast, 200 kPa and 40 °C led to lower flux and to the highest fouling index observed in the experimental design. The center-point replicates also showed good consistency, with mean values of 872.62 L h^−1^ m^−2^ for permeate flux and 87.67% for fouling index, supporting the reliability of the observed trends. Overall, the experimental design indicates that temperature favored permeation only within a pressure range that did not excessively penalize hydraulic stability.

#### 3.1.1. Response-Surface Modeling of the Experimental Data

**Figure 2:**
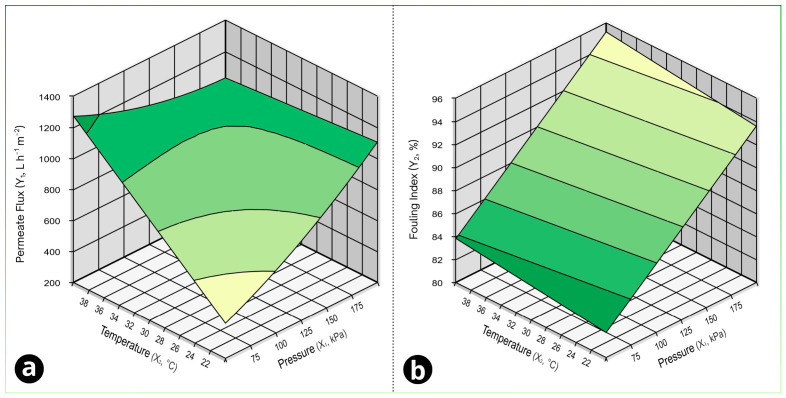
FCD/RSM response surfaces for permeate flux (**a**) and fouling index (**b**).

The statistical analysis confirmed the trends observed in Table 1. According to Table S2 (Supplementary Materials), permeate flux was significantly affected by the linear terms of pressure and temperature, the quadratic term of pressure, and the pressure–temperature interaction. In contrast, the fouling index retained only the linear effects of both variables after reparameterization. This distinction is reflected in Figure 2. In Figure 2a, the curved surface for permeate flux indicates that the response cannot be explained by isolated factor effects, because the effect of temperature depends strongly on the pressure level. By contrast, Figure 2b shows an almost planar surface for the fouling index, indicating a predominantly monotonic increase across the pressure axis.

For permeate flux, the significant interaction term indicates that the gain provided by temperature was not constant throughout the experimental domain. At lower pressure, heating strongly favored permeation, but this benefit weakened at higher pressure, where the additional driving force was no longer efficiently converted into productive flow. This type of behavior is expected in membrane systems when the process shifts from a regime more influenced by bulk transport properties to another more constrained by interfacial resistance and flux limitation [10,11,12,13]. For the fouling index, the predominance of the linear pressure term indicates a simpler statistical behavior, confirming that pressure was the dominant factor controlling the loss of hydraulic performance within the studied range. The mechanistic implications of this trend are discussed in Section 3.2.

The ANOVA results in Table S3 (Supplementary Materials) support the adequacy of the fitted models. Regression was highly significant for both permeate flux and fouling index (*p* < 0.05), with R^2^ values of 0.97 and 0.95, respectively. These values indicate that the models captured the dominant trends of the experimental domain well. However, the significant lack of fit for both responses shows that the quadratic approximation did not fully reproduce all local variations in the system. In practice, this means that the RSM model was robust for identifying the direction of the favorable operating region and the main effects of the variables, but less able to represent the full complexity of the coupled transport behavior. This point helps explain why ANN-based modeling is a relevant complementary approach in the next section.

#### 3.1.2. ANN-Based Representation of the Experimental Responses

**Figure 3:**
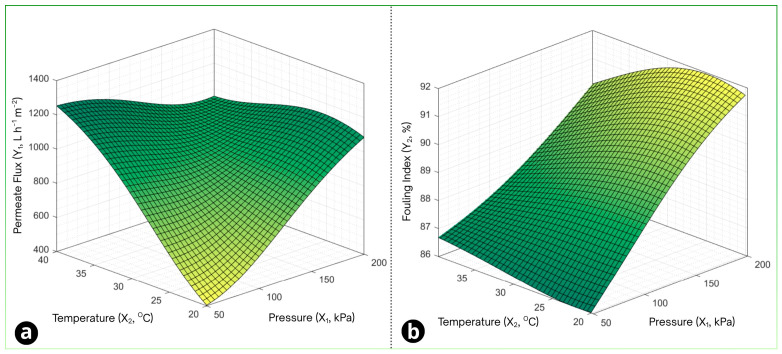
ANN-derived response surfaces for permeate flux (**a**) and fouling index (**b**).

The ANN reproduced the experimental data in Table 1 with small deviations across the sampled pressure–temperature domain, which is consistent with the role of feed-forward neural networks as flexible nonlinear approximators when the response cannot be adequately constrained by a fixed quadratic form [21,24,29,30]. In the present system, this is particularly relevant because permeate flux is governed not only by the applied driving force but also by the balance between convective transport, viscosity effects, concentration polarization, and interfacial resistance buildup, all of which may interact nonlinearly in membrane systems [10,11,23]. Accordingly, ANN-based models can offer an advantage over polynomial regression when the response surface reflects coupled transport effects and local curvature [21,24,29]. This behavior is clearly reflected in Figure 3.

For permeate flux, the ANN surface preserved the strong pressure–temperature coupling already observed experimentally, but represented it with a smoother nonlinear topology than the RSM surface. Temperature exerted a strong positive effect at low pressure, whereas its benefit became progressively attenuated as pressure increased. This pattern is physically coherent with crossflow microfiltration, since heating lowers viscosity and tends to improve mass transfer [9,13], but the gain in permeation is not indefinitely sustained when pressure simultaneously intensifies boundary-layer accumulation and the associated hydraulic penalty [10,11]. For the fouling index, the ANN surface was simpler and remained dominated by pressure, which agrees with the experimental trend that fouling increased monotonically along the pressure axis while temperature played only a secondary role [10,11,12]. Thus, the ANN did not merely fit the data numerically; it reproduced the same qualitative process logic expected for pressure-driven membrane operation [11,23,24].

The post-training diagnostics shown in Figure S4 (Supplementary Materials) reinforce this interpretation. The error histograms for permeate flux and fouling index are centered close to zero, with no evident asymmetry suggestive of marked systematic bias, and the regression plots for training, validation, test, and pooled data all yielded coefficients of determination of 0.99. Taken together, these diagnostics indicate that the selected network learned the input–output relationships of the sampled domain with high internal consistency. This type of result is expected when a compact network is combined with preprocessing and regularization strategies designed to stabilize training and limit unnecessary weight growth, especially for relatively small datasets [30,31]. This internal consistency should also be interpreted in light of the intentionally moderate ANN configuration and repeated-training strategy described in Section 2.2.2, which were adopted to reduce overparameterization risk and improve model stability within the available experimental domain.

At the same time, these statistics must be interpreted within the correct scope. In this manuscript, the 70/15/15 partition was carried out at the sample level, not at the condition level, so replicates from the same pressure–temperature condition could be present in different subsets [24]. Under these circumstances, the ANN diagnostics shown in Figure S4 provide strong evidence of excellent within-domain fit, but they do not by themselves establish strict extrapolative or condition-wise predictive generalization. That distinction matters in membrane-process datasets, where replicate observations from the same operating point are not fully independent for validation purposes. For structured data, blocked or grouped cross-validation is generally more appropriate than purely random splitting when the goal is to estimate performance on unseen conditions, because it reduces information leakage and yields a more realistic assessment of generalization [32].

Therefore, within the scope of this section, the ANN should be interpreted primarily as a flexible nonlinear mapping tool for the experimental domain and as a suitable surrogate model for subsequent GA-based optimization, rather than as a universally more robust predictive model than RSM. Its main scientific contribution in the present study is to represent the local response structure more flexibly than the quadratic model, particularly for permeate flux, whose behavior showed stronger curvature and interaction effects [21,24]. Its practical contribution lies in enabling nonlinear numerical optimization without requiring an explicit polynomial equation and in allowing comparison between two different modeling frameworks within the same experimental domain. Under this interpretation, the ANN adds value not because it replaces FCD/RSM, but because it complements it and helps test whether a data-driven nonlinear framework converges to the same favorable operating region. The stricter assessment of predictive robustness across withheld operating conditions remains better represented by the grouped cross-validation results, where the ANN is evaluated under a more demanding validation structure [32,33].

#### 3.1.3. Comparative Evaluation of the Two Modeling Strategies

Table 5 compares the performance of the FCD/RSM and ANN models using two complementary criteria: sample-level fit and grouped cross-validation across unseen pressure–temperature conditions. This distinction is important because these metrics do not address the same aspect of model performance. While AAD, MSE, RMSE, MAPE, NMSE, NRMSE, and R^2^ describe how closely each model reproduces the experimental observations within the investigated domain, Q^2^ and RMSECV provide a stricter estimate of predictive robustness by evaluating the ability of the models to predict fully withheld pressure–temperature conditions without replicate leakage [34]. This multi-metric interpretation is consistent with current practice in membrane-process modeling, where R^2^ is read together with absolute and normalized error indices rather than used alone as a single measure of model quality [49,50].

At the sample level, the ANN showed a clear advantage for permeate flux, yielding lower errors and a higher coefficient of determination than the corresponding FCD/RSM model. This result indicates that the neural network described the local response structure of flux more efficiently within the sampled domain, which is consistent with the known ability of ANN models to accommodate nonlinear behavior and coupled transport effects in membrane processes [22,49,51]. However, in the present study, this result should be interpreted as evidence of improved within-domain representation rather than as proof of broader predictive superiority. Its scientific significance lies in showing that the permeation response contains sufficient local nonlinearity for a data-driven model to capture features that are only approximated by a quadratic surface. Its practical significance lies in providing a flexible response map for GA-based optimization and in offering an independent framework for comparison with FCD/RSM within the same operating domain.

The fouling index, however, shows the opposite trend. In this case, the FCD/RSM model produced lower errors and a substantially higher R^2^ than the ANN, indicating that this response followed a more regular structure within the investigated domain and was adequately represented by the polynomial model. Therefore, the results suggest that the benefit of ANN was response-dependent rather than universal. This does not diminish the value of the neural-network approach; rather, it indicates that its main strength in the present system was associated with the response that exhibited the more complex nonlinear behavior, namely, permeate flux. Similar contrasts between ANN and polynomial models have been reported in membrane studies, where the superiority of one approach over another depends on the degree of curvature, interaction, and response irregularity present in the dataset [22,51].

A more demanding comparison emerges from the grouped cross-validation results. Under the leave-one-condition-out scheme, the FCD/RSM model showed higher Q^2^ and lower RMSE_CV_ for both responses, indicating greater condition-wise predictive stability. For permeate flux, this contrast was especially pronounced: despite the excellent sample-level fit of the ANN, its cross-validated predictivity was lower than that of the FCD/RSM model. This result should not be interpreted as a contradiction, but rather as evidence that internal fit and external condition-wise predictivity are different properties of the model. In practical terms, the ANN was highly effective for describing the experimental domain and capturing the local topology of the response surface, whereas the FCD/RSM model proved more stable when the prediction task was shifted to fully unseen operating conditions [34,49,50].

From an engineering standpoint, these results indicate that ANN–GA should not be interpreted here as a globally superior alternative to FCD/RSM. With only two operating variables and a relatively small experimental design, the quadratic model already captured the dominant tendencies of the system and showed greater predictive stability under grouped cross-validation, particularly when predicting unseen pressure–temperature conditions. Under these circumstances, the main contribution of ANN was not to replace FCD/RSM but to provide a complementary nonlinear mapping of the response surface, especially for permeate flux, and to serve as the surrogate model for GA-based optimization. In this sense, the value of ANN in the present study is not limited to statistical fit but also includes its role as a nonlinear interpretive and optimization framework that helps reveal whether the selected operating region is robust across different modeling assumptions. Thus, the two approaches are better interpreted as complementary rather than competing, which is also consistent with broader optimization frameworks for pressure-driven membrane systems that integrate empirical and machine-learning models according to the purpose of prediction and optimization [52]. The fact that both approaches converged to the same favorable operating region is therefore an important result in itself, because it strengthens confidence in the selected condition through methodological agreement rather than through the isolated superiority of one model. This convergence strengthens confidence in the selected pressure–temperature region and provides a consistent basis for the next step (Section 3.1.4), in which the predicted values at the selected operating point are compared with the experimental validation results.

#### 3.1.4. GA-Guided Optimization and Validation of the Selected Setpoint

**Figure 4:**
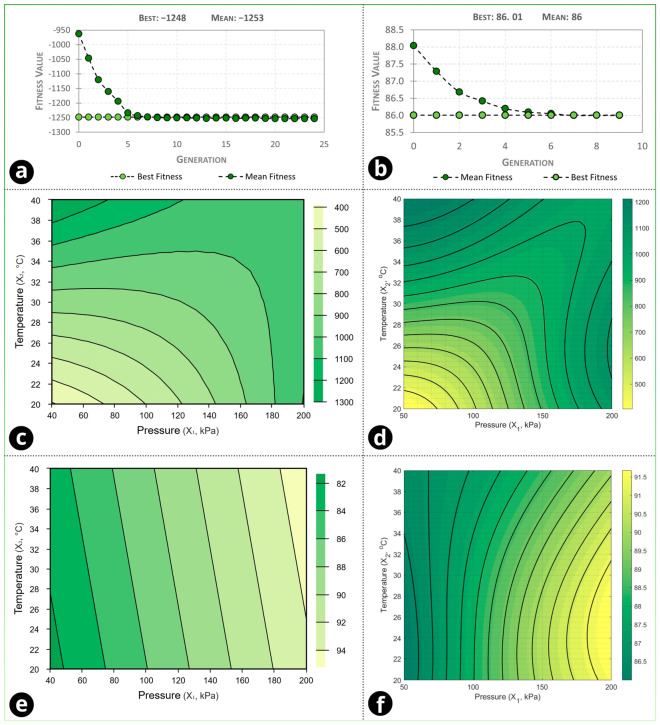
Genetic-algorithm convergence profiles for permeate flux (**a**) and fouling index (**b**), together with contour maps of the corresponding responses predicted by RSM (**c**,**e**) and ANN (**d**,**f**).

The GA runs converged rapidly for both responses, with the best and mean fitness values stabilizing within the first generations (Figure 4a,b). For permeate flux, the negative fitness values reflect the maximization strategy adopted for the ANN output, so the best value corresponds to the highest predicted flux rather than to a negative physical response. For the fouling index, the convergence profile was similarly stable, indicating that the ANN-generated surface was sufficiently regular to support efficient numerical optimization. The balance between flux maximization and fouling minimization was established afterward from the overlap between the favorable regions indicated by the FCD/RSM and ANN contour maps, followed by experimental validation at a practical compromise setpoint. In this sense, the selected condition was not the isolated optimum of a single aggregated objective function, but the operating point judged most attractive when both responses were interpreted together.

This interpretation is reinforced by the contour maps in Figure 4c–f. Both methodologies indicate that the most attractive region is located away from the high-pressure corner of the domain and toward an intermediate pressure–temperature zone, where relatively high permeate flux can be obtained without a proportional increase in fouling. This is particularly important in membrane systems, where the most useful operating condition is rarely the absolute optimum of a single response. Maximizing flux alone may accelerate resistance buildup, whereas minimizing fouling alone may reduce productivity. Thus, the main contribution of the optimization step was not merely the numerical identification of response extrema, but the zone in which both responses remained technically favorable.

Recent analyses of membrane fouling prediction have shown that mathematical and artificial-intelligence models can play complementary roles in membrane-process modeling, with AI-based approaches being particularly useful for representing complex nonlinear relationships, provided that their predictions are interpreted within the limits of the available experimental domain and experimentally validated [50,53].

Experimental validation at 60 kPa and 35 °C confirmed that this selected condition belonged to a favorable operating window. As shown in Table 5, the experimental permeate flux reached 1085.23 ± 23.12 L h^−1^ m^−2^, while the FCD/RSM and ANN predicted 1045.23 ± 20.68 L h^−1^ m^−2^ and 1102.6 L h^−1^ m^−2^, respectively. For the fouling index, the experimental value was 83.56 ± 1.56%, compared with 84.15 ± 0.32% for FCD/RSM and 86.7% for ANN. Although the experimental fouling index at this point (83.56 ± 1.56%) cannot be interpreted as low fouling in an absolute sense, it was acceptable within the practical scope of the present optimization because it was associated with high permeate flux and, as discussed in Section 3.2, with predominantly reversible hydraulic losses rather than severe irreversible membrane damage. Therefore, the selected condition should be interpreted as an operational compromise region rather than as a low-fouling condition. Therefore, both models correctly identified a technically suitable operating region, but with different strengths: the ANN provided the closer point prediction for permeate flux, whereas the FCD/RSM model was more accurate for fouling index.

This response-dependent behavior is fully consistent with the statistical comparison in Table 6, where ANN showed better sample-level performance for the more nonlinear permeation response, while the quadratic model remained more stable for fouling index-related prediction and grouped cross-validation.

From an engineering perspective, this result should be interpreted as complementarity rather than model replacement. In the present two-factor design, FCD/RSM provided a more stable predictive framework under grouped validation, whereas ANN–GA contributed as a flexible nonlinear mapping and optimization tool. Therefore, the practical value of ANN–GA in this study lies less in demonstrating superiority over FCD/RSM and more in showing that an independent nonlinear structure leads to the same favorable operating region. Thus, the selected conditions of 60 kPa and 35 °C can be interpreted as a practically relevant compromise between productivity and hydraulic stability. This validation gives practical support to the modeling strategy and establishes a consistent basis for the subsequent discussion of process performance under the chosen operating condition.

Direct quantitative comparison with previous coconut-water microfiltration studies is limited by differences in membrane material, operating mode, and reporting criteria. Even so, the present results are consistent with previous studies showing that coconut-water clarification under mild pressure conditions can preserve product quality while maintaining attractive permeation performance [17,18,26]. At the same time, the current data make explicit that a favorable productivity window may still be accompanied by a substantial hydraulic penalty, which is why the selected condition should be interpreted as a compromise between high flux and manageable fouling rather than as a low-fouling condition.

### 3.2. Flux Decline, Hydraulic Resistance, and Fouling Behavior

**Figure 5:**
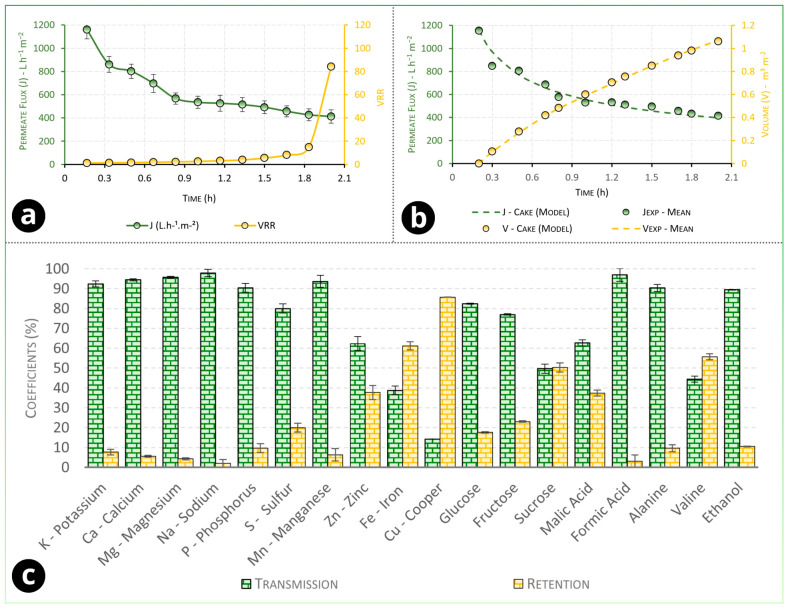
(**a**) Permeate-flux behavior during concentration (*VRR* > 1); (**b**) Cake-filtration model fitting; (**c**) and transmission/rejection of minerals and primary metabolites.

The mean trend shown in Figure 5 indicates a pronounced decline in permeate flux during the initial stage of concentration, followed by a slight and more gradual decrease as filtration progressed. This behavior is typical of constant-pressure crossflow microfiltration of complex liquid foods, in which the early stage is dominated by the rapid establishment of concentration polarization and deposition at the membrane surface, whereas the later stage reflects the progressive consolidation of the external fouling layer and stabilization of the hydraulic regime [10,12]. This interpretation is consistent with the hydraulic permeance data in Table S4, which show a sharp decrease from L_0_^P^ = 2.75 × 10^−8^ to L_1_^P^ = 0.26 × 10^−8^ mPa^−1^ s^−1^ during operation. After physical cleaning, the permeance recovered only to L_2_^P^ = 0.36 × 10^−8^ mPa^−1^ s^−1^, whereas chemical cleaning restored it to L_3_^P^ = 2.56 × 10^−8^ mPa^−1^ s^−1^, corresponding to approximately 96% recovery of the initial clean-water permeance. Therefore, although the process imposed a severe hydraulic penalty during filtration, most of this loss was recoverable after chemical cleaning, indicating that the dominant fouling was largely removable rather than permanently damaging to the membrane.

From a practical standpoint, this high-permeability recovery is desirable. Although the cleaning protocol was defined from preliminary tests and manufacturer guidance, the cleaning frequency and long-term cyclic operation were not systematically investigated in the present study. Even so, recovery above 95% suggests that repeated operation with periodic CIP may be feasible without rapid accumulation of irreversible permeability loss. This result indicates that, under the conditions evaluated here, the main operational challenge is likely to be cleaning management rather than rapid permanent membrane deterioration.

The resistance partition shown in Table S4 reinforces this interpretation. The total resistance reached R_T_ = 3.86 × 10^12^ m^−1^, while the intrinsic membrane resistance accounted for only R_m_ = 0.36 × 10^12^ m^−1^, or 9.38% of the total. By contrast, R_C_ = 1.11 × 10^12^ m^−1^ represented 28.65% of R_T_, and the overall fouling resistance corresponded to 62.02% of the total hydraulic resistance. More importantly, this fouling contribution was dominated by the reversible fraction, since R_R_ = 2.37 × 10^12^ m^−1^ and R_i_ = 0.03 × 10^12^ m^−1^. Thus, the dominant resistances were associated with concentration polarization and reversible deposit buildup at the membrane surface, whereas irreversible resistance remained negligible. The reported fouling index of 90.60% is fully coherent with this result, because it reflects the strong loss of permeance during operation, even though most of that loss was not permanent. In practical terms, the process was strongly fouling-prone during concentration mode, but the prevailing fouling was mainly external and reversible, which is consistent with the typical behavior of fruit-derived liquids processed by pressure-driven membranes [10,12].

This distinction is also important for interpreting the flux profile in Figure 5. The flux profile suggests two distinct stages of fouling. The sharp decline at the beginning of filtration indicates rapid concentration polarization and deposit formation on the membrane surface. The slower decrease observed afterward points to gradual growth and compaction of an external foulant layer, rather than continuous blockage inside the membrane structure [12]. In coconut water, this surface deposit was probably formed by suspended solids and colloidal material naturally present in the beverage. These materials may include aggregates containing polysaccharides and proteins, along with other poorly soluble organic compounds that accumulate close to the membrane under concentration polarization [10,11,12,17]. The selective retention observed for some minerals and metabolites supports this interpretation, since it suggests that the polarized/cake layer may have contained mineral–organic associations and not only inert particles.

This behavior is more favorable than severe irreversible fouling from a process perspective. It indicates that the main performance loss during concentration was caused by hydrodynamic and interfacial effects related to the feed, rather than by permanent deterioration of the membrane. Under the selected condition, productivity was therefore limited mainly by a secondary hydraulic barrier formed during filtration, not by intrinsic damage to the membrane.

Figure 5c also shows that the selected microfiltration condition preserved a large part of the dissolved fraction of coconut water, although the behavior differed among analytes. The major minerals, especially K, Ca, Mg, Na, P, and Mn, showed high transmission and low retention. This indicates that the clarified fraction remained close to the raw beverage in its main electrolyte profile. This result is expected in microfiltration because dissolved ions are much smaller than the nominal membrane pores and are not directly rejected by a clean membrane. Some elements, however, showed lower transmission and higher apparent retention, mainly Cu and Fe and, to a lesser extent, Zn and S. This suggests partial association with colloidal or suspended material and/or interaction with the polarized layer formed during filtration. Thus, the surface layer responsible for flux decline also acted as a dynamic secondary membrane and changed the apparent selectivity of the process. A similar behavior was observed for the primary metabolites. Glucose, fructose, alanine, ethanol, and formic acid were mostly transmitted, whereas sucrose, malic acid, and especially valine had more limited passage and higher retention. Therefore, the process did not act as a simple size-based open filter for all dissolved compounds. Instead, it preserved the main nutritional fraction while selectively retaining compounds more prone to colloidal association, adsorption, or partitioning within the external fouling layer. From a product-quality perspective, this result is relevant because the selected condition maintained much of the characteristic mineral and metabolite profile of coconut water while still providing clarification.

The constant-pressure fouling models were compared using both the agreement with the filtration profile and complementary fit criteria. These criteria included RMSE_V_, R^2^_V_, AIC_CV_, ΔAIC_CV_, RMSE_J_, and R^2^_J_, as reported in Table S5. Among the single constant-pressure models, cake filtration clearly provided the best statistical description of the process, with the lowest AIC_CV_ (−115.043), ΔAIC_CV_ = 0, the lowest RMSE_V_ (0.007 m), and the best diagnostic flux fit, with RMSE_J_ = 43.727 L h^−1^ m^−2^ and R^2^_J_ = 0.956. All other single-mechanism models performed worse, particularly complete blocking, which showed ΔAIC_CV_ = 41.259 and R^2^_J_ = 0.632. Intermediate blocking was the best non-cake alternative, but it remained clearly inferior to cake filtration in both information-theoretic and diagnostic terms. These results indicate that the dominant mechanism governing flux decline under the selected operating condition was the formation of an external cake or gel-like layer rather than progressive internal pore occlusion. This interpretation is consistent with classical constant-pressure fouling theory, in which cake buildup becomes dominant when particle accumulation at the membrane surface overtakes pore-scale obstruction as the main source of hydraulic resistance [41,42].

The combined models confirm the same mechanistic picture. Although cake-complete, cake-intermediate, and cake-standard also showed very good fits, their second fitted parameters collapsed to values essentially equal to zero (K_b_ ≈ 10^−14^; K_i_ ≈ 10^−14^; K_s_ ≈ 10^−12^), while their ΔAIC_CV_ = 2.933 remained higher than that of the single cake model. This means that adding a second fouling mechanism did not produce a meaningful improvement after penalization for model complexity. Therefore, the single cake-filtration model remained the most parsimonious and best-supported description of the data. Even if early pore-related events may have occurred at the beginning of the run, the overall filtration behavior was governed predominantly by external cake-layer development. This affirmation is directly consistent with the resistance analysis, which already showed that reversible surface-associated resistances dominated over irreversible contributions, and also agrees with combined-model analyses reported for membrane fouling in biological and food-related systems [11,42]. Accordingly, cake-layer formation was identified as the dominant fouling mechanism based on the combined analysis of model fit and resistance distribution, rather than on a single statistical parameter.

Taken together, Figure 5 and Tables S4 and S5 define a coherent mechanistic picture of the selected operating condition. The optimization discussed in the previous section successfully identified a productive operating region, but once the process advanced under concentration mode, the dominant limitation became the buildup of a reversible external resistance layer. Thus, the selected condition should not be interpreted as low-fouling in an absolute sense, since the fouling index was high (90.60%), but rather as operationally favorable because the associated fouling was mainly reversible and the membrane retained a high degree of permeability recovery after cleaning. For engineering design, this finding indicates that scale-up should give priority to limiting concentration polarization and cake-layer buildup through control of the flow conditions and cleaning strategy, rather than assuming severe irreversible damage to the membrane. It also connects the optimized operating region, the experimental validation, and the fouling behavior observed during filtration. The selected condition was useful not because fouling was absent, but because productivity remained high and most of the performance loss was reversible, which makes it more manageable for process design.

## 4. Conclusions

This study showed, first, that pressure and temperature did not affect coconut water microfiltration in the same way. Permeate flux responded to a coupled and clearly nonlinear interaction between both variables, whereas the fouling index was governed mainly by pressure. In practical terms, increasing temperature favored permeation only within a pressure range that did not excessively intensify hydraulic losses, confirming that process selection should be based on the balance between productivity and stability rather than on flux maximization alone.

From this experimental basis, the two modeling strategies provided complementary insights, but not equivalent strengths. The FCD/RSM model described the main tendencies of the design space and showed greater predictive stability under grouped cross-validation, especially when the objective was the prediction of unseen operating conditions. The ANN, in turn, represented the permeate-flux response more accurately at the sample level and provided a more flexible nonlinear description of the experimental domain. Therefore, the significance of ANN in the present study lies not in replacing the simpler response-surface approach, but in complementing it through nonlinear response mapping, support for GA-based optimization, and independent convergence toward the same favorable operating region. This complementarity strengthened confidence in the selected pressure–temperature window by showing that the favorable operating region remained consistent across models with different structural assumptions.

This convergence was confirmed experimentally at 60 kPa and 35 °C, where the process achieved 1085.23 ± 23.12 L h^−1^ m^−2^ and 83.56 ± 1.56%. At this point, the ANN yielded the closest prediction for permeate flux, whereas the FCD/RSM model better estimated the fouling index, reinforcing that the two approaches should be interpreted as complementary rather than competing tools for process optimization.

Once the operating condition had been defined, the hydraulic analysis clarified why this region was attractive. During concentration mode, flux decline was severe, but predominantly reversible. The resistance partition showed that the major limitation was associated with concentration polarization and reversible surface deposit buildup, while irreversible resistance remained very small. Fouling modeling led to the same conclusion, with cake filtration providing the best overall description of the process. Thus, the selected condition was favorable not because fouling was absent, but because the dominant hydraulic penalty was largely manageable through cleaning and operational control.

The process was not only hydraulically favorable but also compositionally favorable. The clarified fraction preserved high transmission of the major minerals and relevant primary metabolites, while showing selective retention of some species more closely associated with colloidal material or the polarized layer. Thus, the optimized SiC microfiltration condition combined high permeation performance, acceptable hydraulic stability, strong permeability recovery after cleaning, and substantial preservation of the nutritional profile of coconut water. Taken together, these results support crossflow microfiltration with silicon carbide membranes as a promising laboratory/pilot-scale mild stabilization route for coconut water when both process performance and product-quality retention are considered simultaneously.

It should also be noted that the optimization and modeling results were obtained from a single industrial batch of coconut water. Although this approach was appropriate for controlling raw-material variability within the experimental design, batch-to-batch compositional differences may affect the direct applicability of the fitted models to other production lots. Therefore, the broader applicability of the proposed operating window still depends on further validation under different raw-material lots and on scale-up studies addressing long-term operation, cleaning frequency, membrane lifetime, and industrial-scale economic performance.

## Figures and Tables

**Table 1:** Observed permeate flux and fouling index, with corresponding FCD and ANN predictions, for the studied design conditions.

Exp.	Independent Variables				Dependent Variables					
	Pressure		Temperature		Permeate Flux			Fouling Index		
	(kPa)		(°C)		(L h^−1^ m^−2^)			(%)		
	*X*_1_	*x*_1_	*X*_2_	*x*_2_	*Y*_1_			*Y*_2_		
					Experimental	FCD	ANN	Experimental	FCD	ANN
01	50	−1.0	20	−1.0	403.84 ± 05.89	403.49 ± 23.67	404.82 ± 0.00	80.88 ± 1.30	82.14 ± 0.40	86.027 ± 0.00
02	200	+1.0	20	−1.0	1083.99 ± 72.93	1084.04 ± 23.67	1084.10 ± 0.00	93.83 ± 0.78	93.58 ± 0.40	91.715 ± 0.00
03	50	−1.0	40	+1.0	1247.79 ± 10.97	1250.92 ± 23.67	1253.20 ± 0.00	83.77 ± 0.47	83.81 ± 0.40	86.688 ± 0.00
04	200	+1.0	40	+1.0	1005.54 ± 15.95	1009.06 ± 23.67	1006.70 ± 0.00	95.16 ± 0.46	95.25 ± 0.40	90.034 ± 0.00
05	50	−1.0	30	0.0	890.39 ± 16.95	827.20 ± 14.84	889.51 ± 0.00	84.65 ± 0.44	82.97 ± 0.32	86.266 ± 0.00
06	200	+1.0	30	0.0	1110.54 ± 23.84	1046.55 ± 14.84	1109.80 ± 0.00	94.64 ± 0.32	94.42 ± 0.32	91.669 ± 0.00
07	125	0.0	20	−1.0	733.42 ± 06.84	743.76 ± 14.84	729.10 ± 0.00	88.77 ± 0.25	87.86 ± 0.32	89.354 ± 0.00
08	125	0.0	40	+1.0	1126.60 ± 25.95	1129.99 ± 14.84	1126.20 ± 0.00	89.56 ± 0.34	89.53 ± 0.32	87.949 ± 0.00
09 (c)	125	0.0	30	0.0	883.82	936.88 ± 9.19	870.17 ± 0.00	88.12	88.69 ± 0.20	89.020 ± 0.00
10 (c)	125	0.0	30	0.0	860.23			87.45		
11 (c)	125	0.0	30	0.0	898.15			88.32		
12 (c)	125	0.0	30	0.0	862.34			87.21		
13 (c)	125	0.0	30	0.0	858.56			87.24		

* (x) corresponds to coded factor levels, while (X) refers to the actual experimental settings; (c) indicates the central condition. ** A single homogenized batch was used throughout the study. The experimental matrix comprised nine distinct conditions (eight non-center settings and one center point). The non-center conditions were repeated three times each (*n* = 3), whereas the center condition was repeated five times (*n* = 5; runs 9–13), giving a total of 29 runs. Results are expressed as mean ± SD.

**Table 2:** Hydraulic normalization indicators in a multi-channel SiC membrane (0.6 μm).

Channel Description	*d_h_* (m)	*v_t_* (m s^−1^)	*Re* (–)	*f* (–)	*τ_w_* (Pa)	*Sh* (–)	*k* (m s^−1^)
representative channel; values per channel	0.0040	6.0	2.99 × 10^4^	0.0241	107.7	1084	2.71 × 10^−4^

**Table 3:** Equations adopted for permeance estimation and resistance models in the microfiltration process.

Fouling Mechanisms		Final Model		Unit
$L_{P}^{0}$	Hydraulic permeance coefficient of a clean membrane	LP0=Jw0∆PTM	(14)	mPa^−1^ s^−1^
$L_{P}^{1}$	Hydraulic permeance after filtration	LP1=Jw1∆PTM	(15)	mPa^−1^ s^−1^
$L_{P}^{2}$	Hydraulic permeance after physical washing	LP2=Jw2∆PTM	(16)	mPa^−1^ s^−1^
$L_{P}^{3}$	Hydraulic permeance after chemical washing	LP3=Jw3∆PTM	(17)	mPa^−1^ s^−1^
$R_{T}$	Total system resistance	$R_{T}=R_{M}+R_{C}+R_{F}$	(18)	m^−1^
$R_{M}$	Membrane resistance	RM=1µwLp0	(19)	m^−1^
$R_{C}$	Concentration polarization resistance	RC=1µw(1Lp1−1Lp2)	(20)	m^−1^
$R_{R}$	Reversible resistance	RR=1µw(1Lp2−1Lp3)	(21)	m^−1^
$R_{I}$	Irreversible resistance	RI=1µw(1Lp3−1Lp0)	(22)	m^−1^
$R_{F}$	Fouling resistance	$R_{F}=R_{R}+R_{I\;}$	(23)	m^−1^
F.I.	Fouling index	F.I.=1−Lp1Lp0×100	(24)	%

* “*µ_w_*” represents the viscosity of water (1.0 mPa s), while ΔP_TM_ (kPa) denotes the transmembrane pressure applied in the system [36,37,38].

**Table 4:** Summary of individual and combined models of inlaying under constant pressure.

Fouling Mechanisms	Model’s		Fitted Parameters	Representation
Complete blocking	$V=\frac{J_{0}}{K_{b}}\left(1-e^{-K_{b}t}\right)$	(25)	$K_{b}\;(s^{-1})$	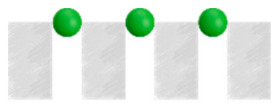
Intermediate blocking	$V=\frac{1}{K_{i}}ln\left(1+K_{i}J_{0}t\right)$	(26)	$K_{i}\;(m^{-1})$	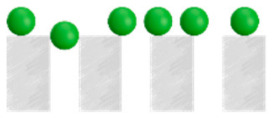
Standard blocking	$V={\left(\frac{1}{J_{0}t}+\frac{K_{s}}{2}\right)}^{-1}$	(27)	$K_{s}\;(m^{-1})$	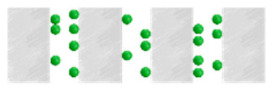
Cake filtration	$V=\frac{1}{K_{c}J_{0}}\left(\sqrt{1+2K_{c}J_{0}^{2}t}-1\right)$	(28)	$K_{c}\;({s\;m}^{-2})$	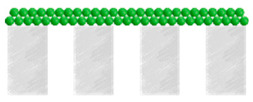
Complete-Standard	$V=\frac{J_{0}}{K_{b}}\left[1-exp\left(-\frac{2K_{b}t}{2+K_{s}J_{0}t}\right)\right]$	(29)	$K_{b}\;\left(s^{-1}\right)$ $K_{s}\;(m^{-1})$	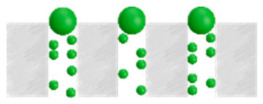
Intermediate-Standard	$V=\frac{1}{K_{i}}ln\left(1+\frac{2K_{i}J_{0}t}{2+K_{s}J_{0}t}\right)$	(30)	$K_{i}\;(m^{-1})$ $K_{s}\;(m^{-1})$	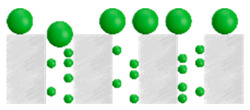
Cake-Complete	$V=\frac{J_{0}}{K_{b}}\left\{1-exp\left[-\frac{K_{b}}{K_{c}J_{0}^{2}}\left(\sqrt{1+2K_{c}J_{0}^{2}t}-1\right)\right]\right\}$	(31)	$K_{c}\;\left({s\;m}^{-2}\right)$ $K_{b}\;(s^{-1})$	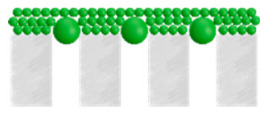
Cake-Intermediate	$V=\frac{1}{K_{i}}ln\left\{1+\frac{K_{i}}{K_{c}J_{0}}\left(\sqrt{1+2K_{c}J_{0}^{2}t}-1\right)\right\}$	(32)	$K_{c}\;\left({s\;m}^{-2}\right)$ $K_{i}\;(m^{-1})$	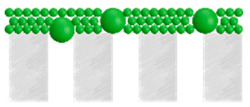
Cake-Standard	$V=\frac{2}{K_{s}}\left[\beta cos\left(\frac{2\pi }{3}-\frac{1}{3}arccos\left(\alpha \right)\right)+\frac{1}{3}\right]$ $\alpha =\frac{8}{27\beta^{3}}+\frac{4K_{s}}{3\beta^{3}K_{c}J_{0}}-\frac{4K_{s}^{2}t}{3\beta^{3}K_{c}}\;\;\;\;\;\;\;\;\;\beta ={\left(\frac{4}{9}+\frac{4K_{s}}{3K_{c}J_{0}}+\frac{2K_{s}^{2}t}{3K_{c}}\right)}^{1/2}$	(33)	$K_{c}\;\left({s\;m}^{-2}\right)$ $K_{s}\;(m^{-1})$	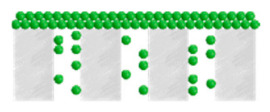

* V is the specific volume of permeate processed per membrane area ($m^{3}\;m^{-2}$, equivalent to m); t is time (h); $J_{0}$ is the initial flux (L h^−1^ m^−2^); $K_{b}$, $K_{i}$, $K_{s}$ e $K_{c}$ are the adjusted constants for complete blocking, intermediate blocking, standard blocking, and cake filtration, respectively. ** All equations in the tables are for constant-pressure operation. *** The Cake-Standard model equation was written in its complete form, without the use of the auxiliary terms alpha and Beta; it results from the direct substitution of these auxiliary variables in the explicit solution published by Bolton [42]. **** When one mechanism dominates, the combined models reduce to the corresponding individual models. m^3^ m^−2^.

**Table 5:** Statistical performance metrics obtained for the experimental design using the evaluated modeling approaches.

**Face Centered Design—FCD**									
**Dependent Variables**	***MAD***	***MSE***	***RMSE***	***MAPE*** **(%)**	***NMSE***	***NRMSE***	***R*** **^2^**	***Q*** **^2^**	***RMSE_CV_***
Permeate Flux	35.36	2109.23	45.93	3.73	2.25	0.05	0.96	0.85	89.85
Fouling Index	0.76	1.00	1.00	0.88	0.01	0.01	0.95	0.70	2.55
**Artificial Neural Network—ANN**									
**Dependent Variables**	***MAD***	***MSE***	***RMSE***	***MAPE*** **(%)**	***NMSE***	***NRMSE***	***R*** **^2^**	***Q*** **^2^**	***RMSE_CV_***
Permeate Flux	15.81	552.60	23.51	1.63	0.59	0.03	0.99	0.57	153.65
Fouling Index	2.51	8.84	2.97	2.84	0.10	0.03	0.60	0.61	2.89

Sample-level metrics *MAD*, *MSE*, *RMSE*, *MAPE*, *NMSE*, *NRMSE*, and *R*^2^: these were computed from all individual observations at the replicate level (n = 29), by comparing the experimental response values ($X_{A}$) with the corresponding model estimates ($X_{P}$) for each sample. In these expressions, $X_{M}$ denotes the mean experimental value of the response, and n is the total number of samples. Grouped cross-validation metrics (*Q*^2^ and *RMSE_CV_*): these were obtained through leave-one-condition-out validation across the 9 unique pressure–temperature conditions, with each fold excluding all replicates associated with one operating condition, thereby preventing replicate leakage.

**Table 6:** Model-based validation of the selected operating condition using FCD/RSM and ANN predictions vs. experimental results.

Dependent Variables	Prediction (FCD)	Prediction (ANN) *	Experimental Data
Permeate Flux (L h^−1^ m^−2^)	1045.23 ± 20.68	1102.6	1085.23 ± 23.12
Fouling Index (%)	84.15 ± 0.32	86.7	83.56 ± 1.56

* ANN predictions are reported as point values, and their uncertainty is evaluated using cross-validated error measures (e.g., *RMSE_CV_*) in combination with sample-level residual statistics. Experimental validation condition: 35 °C and 60 kPa.

## Data Availability

The data supporting the findings of this work are contained within the article. Additional information may be requested from the first author (diogo.rocha@posgrad.ufsc.br).

## Supplementary Materials

The following supporting information can be downloaded at: https://www.mdpi.com/article/10.3390/membranes16070221/s1, Figure S1: Arrangement of factorial, axial, and center points in the two-factor face-centered design; Figure S2: Schematic layout of the silicon carbide crossflow microfiltration unit; Figure S3: Representative ^1^H NMR profile of whole coconut water with its identified components; Figure S4: Post-training ANN diagnostics: error distributions for permeate flux (a) and fouling index (b), and regression performance for training (c), validation (d), test (e), and pooled data (f); Table S1: Organic constituents assigned in coconut water across the evaluated clarification conditions; Table S2: Estimated regression coefficients for the experimental responses before and after model reparameterization; Table S3: Analysis of variance for the response variables of the experimental design; Table S4: Hydraulic permeance values, resistance components, and fouling index obtained for coconut-water microfiltration under concentration mode; Table S5: Estimated fouling parameters and goodness-of-fit metrics for constant-pressure filtration models.

## Abbreviations

The following abbreviations are used in this manuscript:

AICc_V_	Corrected Akaike information criterion based on V(t)
AM	Membrane area (m^2^)
ANN	Artificial neural network
ANN–GA	Artificial neural network–genetic algorithm
ANOVA	Analysis of variance
CA	Feed concentration
CAPES	Coordenação de Aperfeiçoamento de Pessoal de Nível Superior (Brazil)
CFMF	Crossflow microfiltration
CIP	Clean in place
COSY	Correlation spectroscopy
CP	Permeate concentration
CV	Cross-validation
D_2_O	Deuterium oxide
D_ab_	Representative solute diffusivity
EDTA	Ethylenediaminetetraacetic acid
FCD	Face-centered design
F.I.	Fouling index
GA	Genetic algorithm
HMBC	Heteronuclear multiple-bond correlation
HNO_3_	Nitric acid
HSQC	Heteronuclear single-quantum coherence
ICP/OES	Inductively coupled plasma optical emission spectrometry
J	Permeate flux
J_0_	Initial permeate flux
J_p_	Permeate flux
K_2_S_2_O_5_	Potassium metabisulfite
K_b_	Complete blocking model constant
K_c_	Cake filtration model constant
K_i_	Intermediate blocking model constant
K_s_	Standard blocking model constant
L_0_P	Hydraulic permeance of the clean membrane
L_1_P	Hydraulic permeance after filtration
L_2_P	Hydraulic permeance after physical washing
L_3_P	Hydraulic permeance after chemical washing
MAD	Mean absolute deviation
MAPE	Mean absolute percentage error
mapminmax	Min–max normalization function
MFF	Multilayer feed-forward
MSE	Mean squared error
NaClO	Sodium hypochlorite
NaOH	Sodium hydroxide
NMR	Nuclear magnetic resonance
NMSE	Normalized mean squared error
NRMSE	Normalized root mean squared error
PRESAT	Pre-saturation pulse sequence
purelin	Linear transfer function
Q^2^	Cross-validated predictive coefficient of determination
R	Retention coefficient
R^2^	Coefficient of determination
R^2^_J_	Coefficient of determination based on J(t)
R^2^_V_	Coefficient of determination based on V(t)
R_C_	Concentration-polarization resistance
Re	Reynolds number
R_F_	Fouling resistance
R_I_	Irreversible resistance
R_M_	Intrinsic membrane resistance
RMSE	Root mean squared error
RMSE_CV_	Cross-validated root mean squared error
RMSE_J_	Root mean squared error based on J(t)
RMSE_V_	Root mean squared error based on V(t)
R_R_	Reversible resistance
RSM	Response surface methodology
R_T_	Total resistance
Sc	Schmidt number
Sh	Sherwood number
SiC	Silicon carbide
SSR_V_	Sum of squared residuals based on V(t)
T	Transmission coefficient
t	Time (h)
tansig	Hyperbolic tangent sigmoid transfer function
TMSP-d_4_	Sodium 3-(trimethylsilyl)propionate-d_4_
trainbr	Bayesian regularization backpropagation training function
v_0_	Initial superficial velocity per membrane area
v_t_	In-channel tangential velocity
V(t)	Specific permeate volume
V_F_	Total feed volume (L)
V_P_	Permeate volume collected (L)
V_R_	Retentate volume (L)
VRR	Volumetric reduction ratio
x_1_	Pressure variable in coded values
x_2_	Temperature variable in coded values
X_1_	Pressure variable in actual values
X_2_	Temperature variable in actual values
Y_1_	Permeate flux response
Y_2_	Fouling index response
ΔAICc_V_	Difference from the lowest AICc_V_
ΔPTM	Transmembrane pressure
μ	Dynamic viscosity
μ_w_	Water viscosity
τ_w_	Wall shear stress
