# Supplementary material for: Coconut Water Microfiltration Optimization Using Response Surface Modeling, Neural Networks, and Genetic Algorithms: Performance and Nutritional Retention
Source: Membranes (Basel). 2026 Jun 26;16(7):221. doi: 10.3390/membranes16070221 (PMC13414442; doi:10.3390/membranes16070221)
Supplement: Supplementary file 1 [file membranes-16-00221-s001.zip › membranes-4322025-supplementary.pdf]

membranes

MDPI

Article

Coconut Water Microfiltration Optimization Using Response Surface Modeling, Neural Networks, and Genetic Algorithms: Performance and Nutritional Retention

José Diogo da Rocha Viana [1], Arthur Claudio Rodrigues de Souza [2], Paulo Riceli Vasconcelos Ribeiro [2], Lorena Mara Alexandre Silva [2], Kirley Marques Canuto [2], Katia Rezzadori [3], Giordana Demaman Arend [1,*], Ana Paula Dionísio [2,*] and José Carlos Cunha Petrus [1]

1 Department of Chemical and Food Engineering, Federal University of Santa Catarina, Florianópolis 88040-900, SC, Brazil; diogo.rocha@posgrad.ufsc.br (J.D.d.R.V.), jose.petrus@ufsc.br (J.C.C.P)
2 Embrapa Tropical Agroindustry, Fortaleza 60511-110, Brazil; arthur.souza@embrapa.br (A.C.R.S), paulo.riceli@embrapa.br (P.R.V.R), lorena.mara@embrapa.br (L.M.A.S), kirley.canuto@embrapa.br (K.M.C)
3 Department of Food Science and Technology, Federal University of Santa Catarina, Av. Ademar Gonzaga, 1346, Itacorubi, Florianópolis 88034-000, SC, Brazil; katia.rezzadori@ufsc.br (K.R.)

* Correspondence:
giordana.darend@gmail.com (G.D.A), ana.dionisio@embrapa.br (A.P.D.)

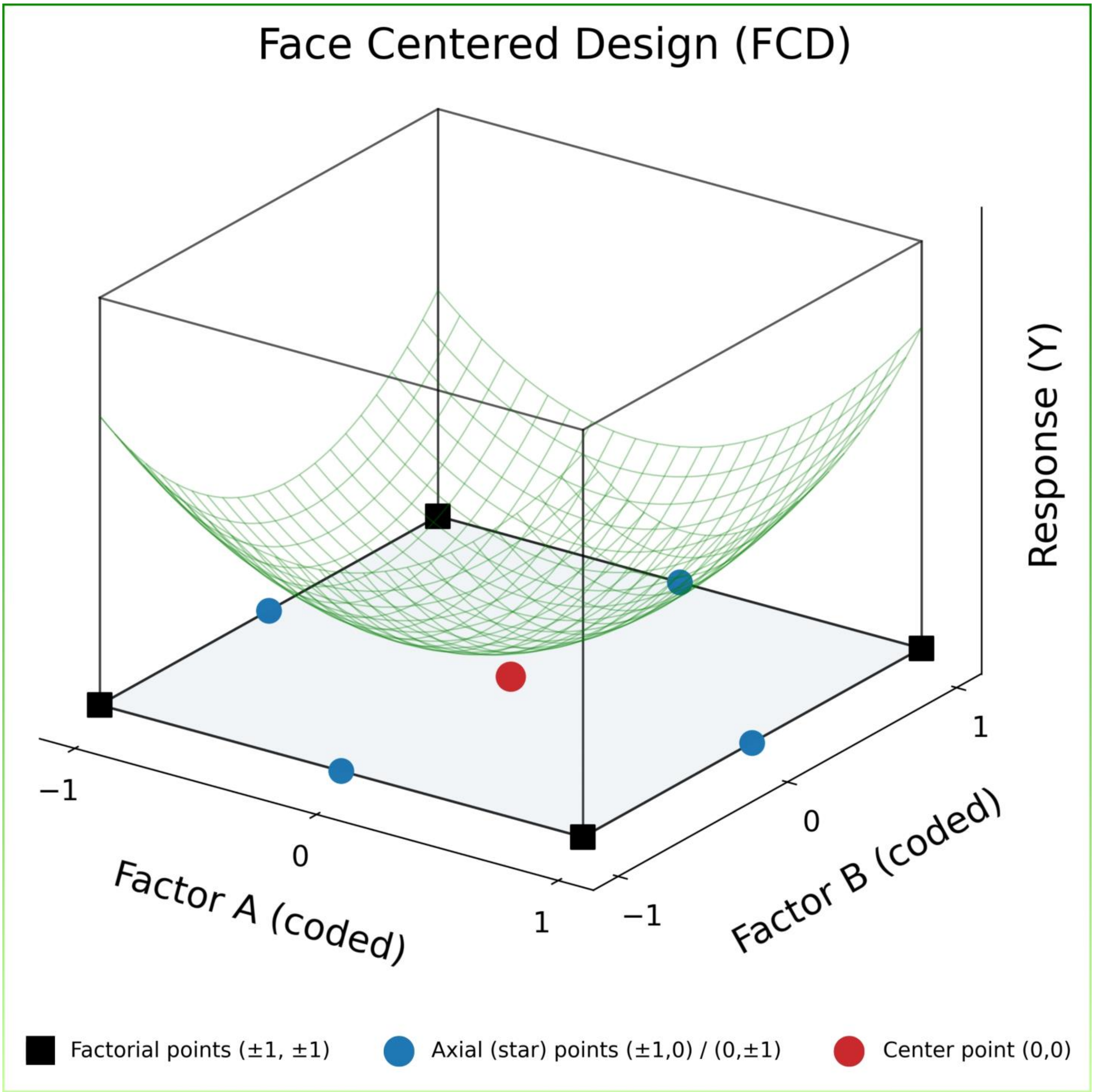

Figure S1. Arrangement of factorial, axial, and center points in the two-factor face-centered design.

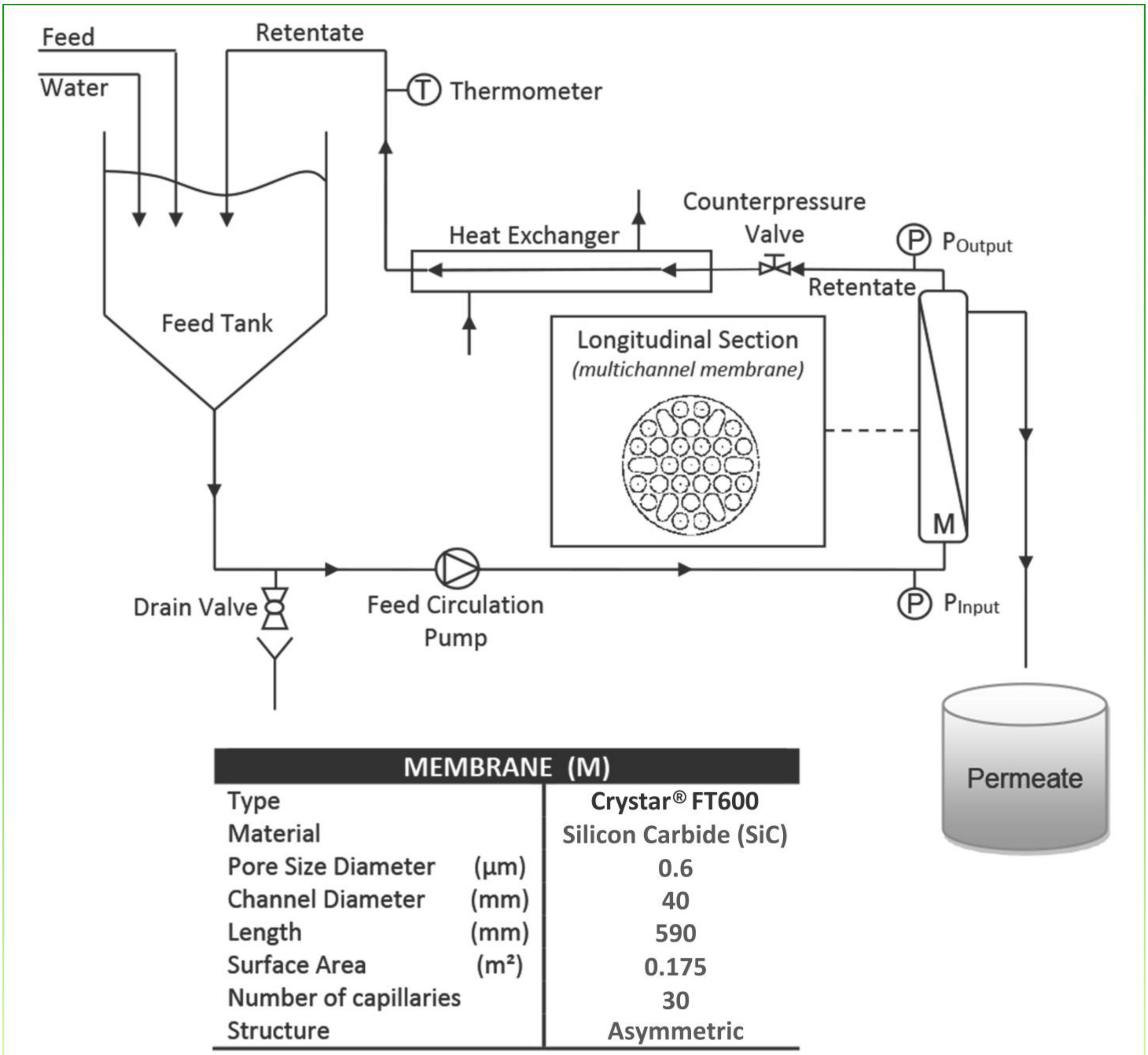

MEMBRANE (M)	
Type	Crystar® FT600
Material	Silicon Carbide (SiC)
Pore Size Diameter (μm)	0.6
Channel Diameter (mm)	40
Length (mm)	590
Surface Area (m^2)	0.175
Number of capillaries	30
Structure	Asymmetric

Figure S2. Schematic layout of the silicon carbide crossflow microfiltration unit.

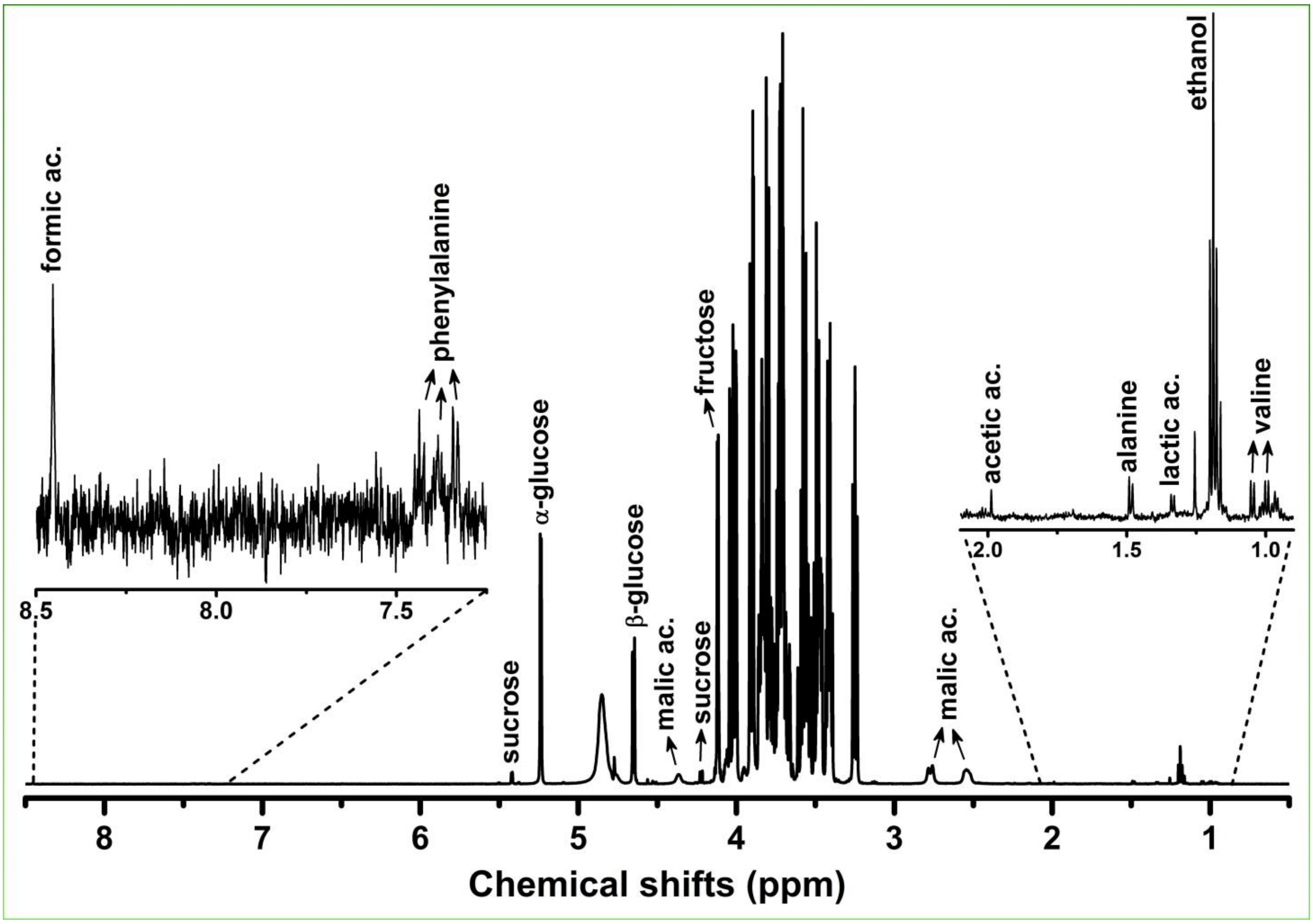

Figure S3. Representative ^{1}H NMR profile of whole coconut water with its identified components.

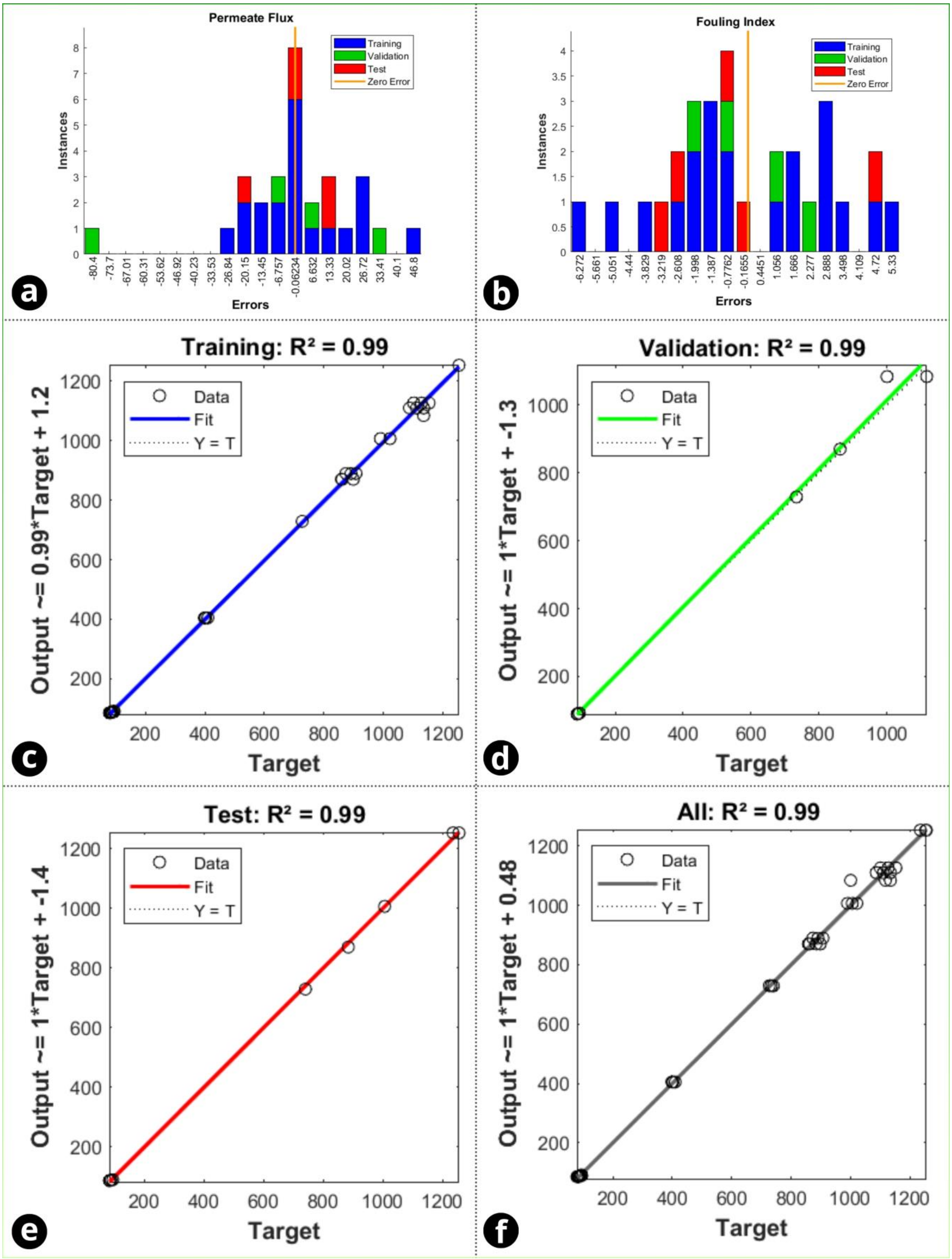

Figure S4. Post-training ANN diagnostics: error distributions for permeate flux (a) and fouling index (b), and regression performance for training (c), validation (d), test (e), and pooled data (f).

Table S1. Organic constituents assigned in coconut water across the evaluated clarification conditions.

Compound / Structure	*δ ¹H (multip.*, J in Hz)*	*δ ¹³C*	*Ref. ¹H*	*Ref. ¹³C*
AMINO ACIDS				
Alanine 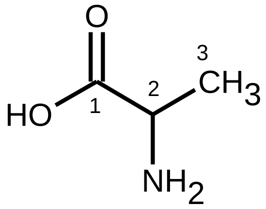	3 – 1.45 (*d* 7.2)<br>2 – 3.89 (o)	17.9<br>53.1	1.52 (*d* 7.30)<br>3.90 (*q* 7.30)	19.1<br>53.4
Phenylalanine 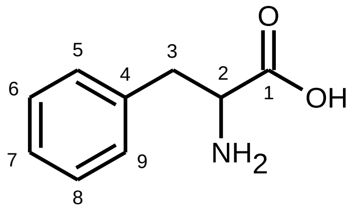	5.9 – 7.42 (*m*)<br>6.8 – 7.48 (*m*)<br>7 – 7.45 (*m*)	132.5<br>132.4<br>130.8	7.32 (*d* 6.98)<br>7.42 (*m*)<br>7.37 (*m*)	132.1<br>131.8<br>130.4
Valine 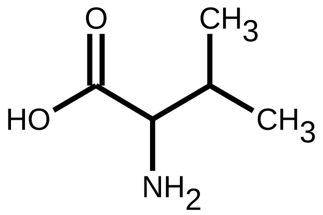	2 – no<br>3 –2.29 (o)<br>4 – 0.98 (*d* 7.2)<br>5 – 1.01 (*d* 7.2)	no<br>32.0<br>19.6<br>20.8	3.82 (*d* 4.4)<br>2.33 (*m*)<br>1.02 (*d* 7.1)<br>1.06 (*d* 7.1)	n<br>32.0<br>19.1<br>20.9
ORGANIC ACIDS				
Acetic Acid 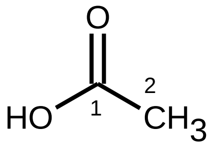	1 – no<br>2 – 1.98 (*s*)	176.9<br>22.4	no<br>2.08 (*s*)	184.1<br>26.0
Formic Acid 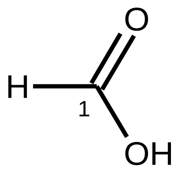	1 – 8.46 (*s*)	no	8.40 (*s*)	172.4
Lactic Acid 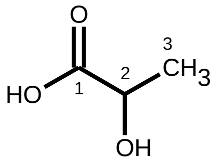	3 – 1.32 (*d* 7.40)<br>2 – 4.40 (o)	22.4<br>69.6	1.37 (*d* 7.20)<br>4.42 (*q* 7.20)	22.9<br>71.4

Malic Acid				
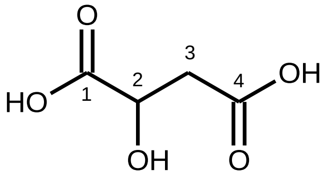	2 – 4.35 (o)	69.3	4.29 (*dd* 10.10, 2.90)	73.2
	3 – 2.58; 2.73 (*m*)	40.3	2.34; 2.65 (*dd* 15.40, 10.10)	45.5
CARBOHYDRATES				
α-glucose	1 – 5.20 (*d* 4.08)	95.6	5.25 (*d* 3.80)	95.4
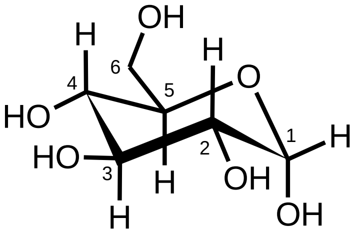	2 – 3.47 (*m*)	72.3	3.89-3.36 (o)	72.2
	3 – 3.77 (*m*)	75.6	n	76.0
	4 – 3.56 (*m*)	74.0	n	72.8
	5 – 3.72 (*m*)	63.9	n	64.2
	6 – 3.85 (*m*)	75.5	n	74.5
β-glucose	1 – 4.61 (*d* 7.80)	99.3	4.66 (*d* 8.10)	99.2
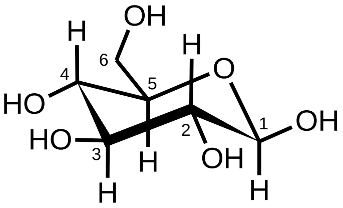	2 – 3.26 (*m*)	77.5	3.25 (*t* 8.40)	77.6
	3 – 3.75 (*m*)	63.6	n	56.1
	4 – 3.48 (*m*)	78.8	n	79.0
	5 – 3.41 (*m*)	72.2	n	72.8
	6 – 3.90 (*m*)	63.7	n	63.1
Fructose	1 – o	o	3.58 (*m*)	65.6
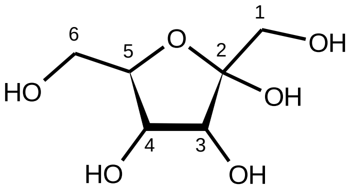	2 – no	107.4	no	104.2
	3 – 4.09 (*dd* 3.55)	77.5	4.11 (*m*)	78.2
	4 – 4.09 (*d* 3.55)	78.5	4.11 (*m*)	77.4
	5 – 3.82 (o)	83.8	3.82 (*m*)	83.6
	6 – 4.02 (o)	66.5	4.01 (*m*)	66.1
Sucrose				
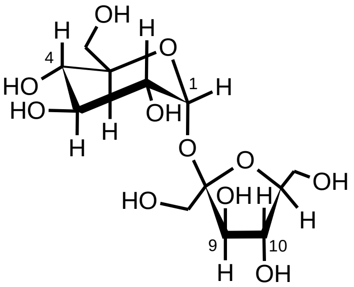	1 – 5.39 (*d* 3.7)	93.8	5.44 (*d* 3.8)	94.7
	9 – 4.20 (*d*)	79.4	4.24 (*d* 9.0)	76.6
OTHER COMPOUNDS				
Ethanol				
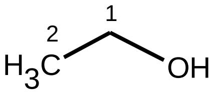	1 – 3.65 (o)	54.5	3.64 (*q* 7.08)	60.3
	2 – 1.18 (*t* 7.20)	21.2	1.17 (*t* 7.08)	19.6

* *s* – singlet; *d* – duplet; *t* – triplet; *q* – quadruplet; *quin* – quintet; *dd* – double duplet; *dt* – double triplet; o – overlapping signal; n – no information; no – not observed.

Table S2. Estimated regression coefficients for the experimental responses before and after model reparameterization.

**Permeate Flux (Y_1, L h^{-1} m^{-2})**					**Fouling Index (Y_2, %)**				
**Factors**	**Regression Coefficient**	**Standard Error**	t_{cal}	*p - value*	**Factors**	**Regression Coefficient**	**Standard Error**	t_{cal}	*p - value*
Average	912,37	15,39	59,28	0,00	Average	85,55	0,39	226,32	0,00
x_1 (L)	109,67	9,87	11,11	0,00	x_1 (L)	5,72	0,25	22,81	0,00
x_1^2 (Q)	54,97	16,14	3,41	0,00	x_1^2 (Q)	0,35	0,41	0,86	0,40
x_2 (L)	193,11	9,87	19,57	0,00	x_2 (L)	0,84	0,25	3,33	0,00
x_2^2 (Q)	-15,48	16,14	-0,96	0,35	x_2^2 (Q)	-0,13	0,41	-0,31	0,76
$x_1.x_2$	-230,60	12,09	-19,08	0,00	$x_1.x_2$	-0,39	0,31	-1,26	0,22
**Significant Coefficients (Reparametrized)**					**Significant Coefficients (Reparametrized)**				
**Factors**	**Regression Coefficient**	**Standard Error**	t_{cal}	*p - value*	**Factors**	**Regression Coefficient**	**Standard Error**	t_{cal}	*p - value*
Average	903,92	12,60	71,73	0,00	Average	88,69	0,20	454,22	0,00
x_1 (L)	109,67	11,66	11,13	0,00	x_1 (L)	5,72	0,25	23,09	0,00
x_1^2 (Q)	53,09	16,00	3,32	0,00					
x_2 (L)	193,11	9,85	19,60	0,00	x_2 (L)	0,84	0,25	3,37	0,00
$x_1.x_2$	- 230,60	12,07	- 19,11	0,00					

* (L) = linear term; (Q) = quadratic term.

Table S3. Analysis of variance for the response variables of the experimental design.

DEPENDENT VARIABLE (Permeate Flux)

Source of variation	Sum of squares	Degrees of freedom	Mean square	F_{test}	*p - value*	R^2
**Regression**	1545156,6	4,0	386289,1	221,1	0,00	
**Residuals**	41924,6	24,0	1746,9			
**Lack of fit**	26083,6	4,0	6520,9	8,2	0,00	0.97
**Pure error**	15840,9	20,0	792,0			
**Total**	1587081,1	28,0				
**Exp. Model**	$Y_1 = 903{,}92 + 109{,}67x_1 + 53{,}09x_1^2 + 193{,}11x_2 - 230{,}60x_1x_2$					(01)

DEPENDENT VARIABLE (Fouling Index)

Source of variation	Sum of squares	Degrees of freedom	Mean square	F_{test}	*p - value*	R^2
**Regression**	602,0	2,0	301,0	272,2	0,00	
**Residuals**	28,7	26,0	1,1			
**Lack of fit**	21,2	6,0	3,5	9,4	0,01	0.95
**Pure error**	7,5	20,0	0,4			
**Total**	630,7	28,0				
**Exp. Model**	$Y_2 = 88{,}69 + 5{,}72x_1 + 0{,}84x_2$					(02)

* $F_{tabuleted\ 4;24}$ = 278; $F_{tabuleted\ 2;26}$ = 3.37; ** Y_1 and Y_2 are permeate flux (L h^{-1} m^{-2}) and fouling index (%), respectively, and x_1 and x_2 refer to pressure (kPa) and temperature (°C), respectively.

Table S4. Hydraulic permeance values, resistance components, and fouling index obtained for coconut-water microfiltration under concentration mode.

L^0_P (10^{-8}) (mPa^{-1} s^{-1})	L^1_P (10^{-8})	L^2_P (10^{-8})	L^3_P (10^{-8})
2.75	0.26	0.36	2.66

R_T (10^{12}) (m^{-1})	R_M (10^{12})	R_C (10^{12})	R_R (10^{12})	R_I (10^{12})	R_M / R_T (%)	R_C / R_T	R_F / R_T	F.I
3.86	0.36	1.11	2.37	0.03	9.38	28.65	62.02	90.60

Table S5. Estimated fouling parameters and goodness-of-fit metrics for constant-pressure filtration models.

Model	Parameters (adjusted)		$RMSE_V$ (m)	R^2_V	$AICc_V$	$\Delta AICc_V$	$RMSE_J$ (L h^{-1} m^{-2})	R^2_J
Single Models								
Complete blocking	$K_b = 2.599e^{-04}$		0.042	0.984	-73.7829	41.259	126.975	0.632
Intermediate blocking	$K_i = 1.21141$		0.021	0.996	-89.8284	25.214	72.4663	0.879
Standard blocking	$K_s = 0.99289$		0.031	0.992	-81.2839	33.759	95.7021	0.791
Cake filtration	$K_c = 5618.81$		0.007	0.999	-115.043	0.000	43.727	0.956
Combined Models								
Complete-Standard	$K_b = 9.27e^{-10}$	$K_s = 0.99286$	0.031	0.992	-78.351	36.692	95.702	0.791
Intermediate-Standard	$K_i = 1.21142$	$K_s = 1.278e^{-09}$	0.021	0.999	-86.895	28.147	72.466	0.879
Cake-Complete	$K_c = 5618.81$	$K_b = 3.232e^{-14}$	0.007	0.999	-112.109	2.933	43.727	0.956
Cake-Intermediate	$K_c = 5618.81$	$K_i = 8.640e^{-14}$	0.007	0.999	-112.109	2.933	43.727	0.956
Cake-Standard	$K_c = 5618.8$	$K_s = 1.069e^{-12}$	0.007	0.999	-112.109	2.933	43.727	0.956

* $V(t)$ is the specific permeate volume ($\mathrm{m^3\ m^{-2}}$), numerically equivalent to meters (m), obtained by time-integration of $J(t)$. Model fitting minimizes SSR_V and reports $RMSE_V$ and R_V^2; $RMSE_J$ and $R_J^2$are provided as diagnostic metrics. $AICc_V$ is computed from SSR_V with a penalty for the number of fitted parameters ($k = 1$ for single-mechanism models; $k = 2$for combined models). $\Delta AICc_V$ is referenced to the lowest $AICc_V$. Parameter units: $K_b(\mathrm{s^{-1}})$, $K_i(\mathrm{m^{-1}})$, $K_s(\mathrm{m^{-1}})$, and $K_c(\mathrm{s\,m^{-2}})$.
